# Cerebral White Matter Hyperintensities in a biracial US cohort: prevalence and risk factors

**DOI:** 10.1002/alz70856_102224

**Published:** 2025-12-25

**Authors:** Russell Sawyer, Oluchi Ekenze, Ya Yuan, Mary Cushman, Virginia J Howard, Suzanne E Judd, George Howard, Joseph Broderick, Rhonna Shatz, Hyacinth I Hyacinth

**Affiliations:** ^1^ University of Cincinnati, Cincinnati, OH, USA; ^2^ Boston University, Boston, MA, USA; ^3^ University of Alabama, Birmingham, AL, USA; ^4^ University of Vermont, Burlington, VT, USA; ^5^ School of Public Health, University of Alabama at Birmingham, Birmingham, AL, USA; ^6^ UAB, Birmingham, AL, USA

## Abstract

**Background:**

White matter hyperintensities (WMH), an important feature of cerebral small vessel disease, are areas of hyperintense signal on T2‐weighted, fluid‐attenuated inversion recovery (FLAIR) Magnetic Resonance Imaging (MRI). WMH are associated with a 25% greater risk of Alzheimer's disease and 73% greater risk of vascular dementia. Few studies have been adequately powered to examine the interplay between race, geographic region, and WMH burden.

**Method:**

Using clinical MRI images from REason for Racial And Geographic Differences in Stroke (REGARDS) participants with stroke/TIA, combined with demographic, clinical and APOE genotype data, we assessed the relationship between race and geographical region with WMH lesion volume in the (1) whole brain, (2) specific brain regions and (3) specific penetrating arteries distribution. WMH lesion volume segmentation was performed using Unidentified Bright Object (UBO) detector. Generalized linear models were used to compare WMH volume in Black vs White participants, further stratified by residence, and in stroke belt vs non‐stroke belt resident, further stratified by race. We also examined whether there is any race x geographical region interaction. The multivariate model was adjusted for relevant covariates.

**Result:**

We analyzed MRI from 1094 REGARDS participants with a stroke/TIA (430 Black participants and 664 White participants). In the fully adjusted model Black participants had greater WMH lesion volumes in for whole brain (β = 0.36, *p*‐value = 2.9×10^‐5^, 95% CI = 0.19 ‐ 0.52), periventricular (β = 0.35, *p*‐value = 9.1×10^‐5^, 95% CI = 0.18‐0.53), and deep (β=0.42, *p*‐value = 3.8×10^‐5^, 95% CI = 0.22 ‐0.61) regions, as well as in 11 additional brain regions and penetrating arterial territories. There was no significant association between geographical region and WMH lesion volume in Black or White participants. Interaction analysis revealed no interaction between race and region in WMH burden.

**Conclusion:**

Among our study sample, after adjusting for demographic, clinical, and genetic factors, Black participants had a significantly more likely to have larger WMH lesion volume compared with White participants. This may explain some of the racial differences in rates of Alzheimer's disease and vascular cognitive impairment. Studies are needed to better understand the mechanisms leading to the development of WMH.

